# Whitewashing

**Published:** 1871-10

**Authors:** 


### WHITEWASHING.

Pour boiling water on unslacked lime, that is, lime in the shape of stones, which have not fallen apart by exposure to the dampness of the atmosphere; cover the vessel over to prevent the steam from carrying away the finest particles of the lime, which are needed to permeate the smallest crevices; add one pint of salt to four gallons of the whitewash, stir it well, and apply it where desired; the salt unites with the lime, and forms a smooth, hard, white surface, lasting next to paint. A good whitewashing of all the fences and outbuildings of a farm-house adds so much to the cheerfulness, tidiness, and healthfulness of the premises, that every intelligent farmer owes it to himself, his family, his neighborhood, and the general interest of the community in which he resides, to make it a point, at least once a year, to use a liberal supply of good whitewash wherever it may be applied with advantage, with the assurance that it will make his place much more salable, besides more healthful.

